# Nanoporous Au Behavior in Methyl Orange Solutions

**DOI:** 10.3390/molecules29091950

**Published:** 2024-04-24

**Authors:** Andrea Pinna, Giorgio Pia, Nicola Melis, Mirko Prato, Maria Giorgia Cutrufello, Elisa Sogne, Andrea Falqui, Luca Pilia

**Affiliations:** 1 Department of Mechanical, Chemical and Materials Engineering, University of Cagliari, Via Marengo 2, 09123 Cagliari, Italy; 2 Materials Characterization Facility, Italian Institute of Technology, Via Morego 30, 16163 Genoa, Italy; 3 Department of Chemical and Geological Sciences, University of Cagliari, S.P. Monserrato-Sestu Km 0.700, 09042 Cagliari, Italy; gcutrufe@unica.it; 4 National Interuniversity Consortium of Materials Science and Technology (INSTM), Via Giuseppe Giusti 9, 50121 Firenze, Italy; 5 PoliFAB, Polytechnic of Milan, Via Giuseppe Colombo, 81, 20133 Milan, Italy; 6 Department of Physics “Aldo Pontremoli”, University of Milan, Via Celoria 16, 20133 Milan, Italy

**Keywords:** nanoporous gold, adsorption, catalysis, methyl orange

## Abstract

Nanoporous (NP) gold, the most extensively studied and efficient NP metal, possesses exceptional properties that make it highly attractive for advanced technological applications. Notably, its remarkable catalytic properties in various significant reactions hold enormous potential. However, the exploration of its catalytic activity in the degradation of water pollutants remains limited. Nevertheless, previous research has reported the catalytic activity of NP Au in the degradation of methyl orange (MO), a toxic azo dye commonly found in water. This study aims to investigate the behavior of nanoporous gold in MO solutions using UV-Vis absorption spectroscopy and high-performance liquid chromatography. The NP Au was prepared by chemical removal of silver atoms of an AuAg precursor alloy prepared by ball milling. Immersion tests were conducted on both pellets and powders of NP Au, followed by examination of the residual solutions. Additionally, X-ray photoelectron spectroscopy and electrochemical impedance measurements were employed to analyze NP Au after the tests. The findings reveal that the predominant and faster process involves the partially reversible adsorption of MO onto NP Au, while the catalytic degradation of the dye plays a secondary and slower role in this system.

## 1. Introduction

Nanoporous metals (NPMs) belong to a class of materials with unique properties related to their peculiar nanostructured morphology. They present a spontaneous disordered three-dimensional open-cell structure (bicontinuous network) composed of ligaments and massive nodes. The resulting pores, as well as the ligaments, possess characteristic dimensions ranging from 2 to 100 nm, which collectively contribute to a structure with a significant specific surface area [1,2,3,4,5,6].

Different methods are used to fabricate NP metals [7,8,9,10,11]. However, primarily, they are obtained through the process of chemical or electrochemical de-alloying, fundamentally relying on the preferential and selective dissolution in an acidic solution of one or more elements from a parent alloy. During this process, individual atoms or small clusters thereof transition into solution, while a concurrent reconstruction of solid–liquid interfacial zones occurs, governed by surface mass transport phenomena.

Within the residual alloy, a connected porosity progressively develops, giving rise to a complex network of relatively thin ligaments interconnected by nodes where a greater mass concentration is observed. The volumetric shrinkage resulting from dissolution induces internal stresses that can be mitigated through thermal treatments. These treatments, depending on various factors, such as the characteristic dimensions of the ligaments, the temperature applied during the thermal treatment, and the exposure time to treatment, can lead to a coarsening of the structural parts (nodes and ligaments) [12,13,14].

However, despite its apparent simplicity, de-alloying presents several limitations. For instance, while it works optimally for the production of NP Au, the same cannot be said for the fabrication of non-noble NP metals, significantly limiting its fields of application. Moreover, conducting the process in an aqueous environment precludes the use of metals that are easily oxidizable. Finally, the wide array of variables at play during the process makes it difficult to fully control the experimental phase and, consequently, the outcome, especially at the atomic scale.

Consequently, the pursuit of novel synthetic pathways for NP metals stands as a critical advancement. The meticulous examination of the morphology characterizing pores, ligaments, and nodes is a pivotal step towards validating and assessing the adaptability of manufacturing techniques. Achieving control over structural characteristics through sophisticated material manipulation on the nanoscale is a key goal for unlocking the full potential of NP metal foams. Furthermore, a deeper understanding of the complex interplay between structure and properties paves the way for the intelligent design of NP metals [15,16].

Despite numerous challenges, the unique structural morphology of nanoporous metals marks a considerable innovation, endowing these materials with distinct physical and chemical properties. This advancement not only fuels intriguing inquiries into the fundamental relationships between structure and function but also showcases their substantial potential across diverse scientific and technological domains. The array of applications extends from structural materials and catalysis to sensing and energy-oriented electrochemistry, illustrating the versatility and impact of nanoporous metals in advancing current and future innovations.

Among NPMs, nanoporous gold (NP Au) is by far the most investigated material. As for the majority of NPMs, NP Au is also usually obtained by chemical or electrochemical dealloying, which leads to the etching of the less noble metal (silver in most cases). Indeed, in the scientific literature, it is possible to find papers which report the use of NP Au as material for (a) new generation of high sensitivity optical and electrochemical sensors to detect biological molecules or pollutants; (b) medical treatment and diagnostics in controlled drug delivery, hyperthermia treatment for cancer; (c) surface-enhanced Raman scattering, improving the capability to find chemical substances and biomarkers; (d) the phototermal and photocalysis field, using the capacity of NP Au to convert light into heat for activating chemical reactions; (e) catalysis in different reactions, including air purification and the production of specific fine chemicals [10,17,18].

In particular, regarding the potential applications mentioned above, several papers have reported the use of NP Au as a catalyst [19,20,21]. Indeed, the interest in gold-based systems is related to the nontoxicity of this metal and to its capability to favor selective reactions in mild conditions. NP Au has been investigated as a catalyst in CO oxidation at low temperature and pressures [22,23,24,25], as well as in the gas phase oxidative coupling of methanol at relative low temperature [20,26]. In the last decades, great attention has been paid to nanosized Au catalysts, supported on CeO_2_ [27], TiO_2_ [28], carbon [29] or polymers [30], for reactions of selective oxidation of alcohols [27,28] or hydrocarbons [29]. However, the monolithic form of NP Au allows to overcome the need of a support, which is instead necessary in the case of nanoparticles to enable an easier recovery of the catalyst from the reaction environment [31].

A few years ago, Hakamada et al. proposed use of an NP Au monolith as a catalyst for methyl orange (MO) degradation in solution, at room temperature and in the dark [32] MO is a molecule which belongs to the class of azo dyes (R-N=N-R’); this family of compounds is widely employed in the textile industry because of its intense color and its stability [33]. Furthermore, MO has also been used as a probe for tests in photocatalytic studies devoted to the degradation of this class of organic dyes, which are pollutants and, therefore, damage the natural environment, especially water. Because of the very large use of synthetic dyes in industry, their wastewater pollution is considered one of the most important problems for the environment [34]. For this reason, many efforts are being made to find methods to remove these pollutants. In addition to the investigations concerning the adsorption technique [35,36,37,38,39], many studies have focused on dye degradation. In particular, in the cases of heterogenous semiconductor photocatalysts, holes or electrons react with water or oxygen to form several oxygen-containing species, such as hydroperhydroxyl, superoxide and hydroxyl radicals, which, thanks to their high reactivity as oxidizing agents, cause the degradation of the dyes [40,41,42,43,44].

In the present work, it is shown that in the presence of monolithic NP Au, only a partial degradation of MO occurs, whereas most of the dye is adsorbed onto the NP Au surface.

Our study was carried out by means of ultraviolet–visible (UV-Vis), energy dispersive X-ray (EDS), and X-ray photoelectron (XPS) spectroscopies, high-performance liquid chromatography (HPLC), scanning electron microscopy (SEM) and impedance measurements.

## 2. Results and Discussion

Au in NP form was fabricated by the chemical etching of a cold-pressed pellet of an AuAg alloy using nitric acid at 70% as the corrosive agent for 24 h. The starting alloy was obtained by ball milling gold and silver powders in the atomic ratio of 3:7.

**Figure 1:**
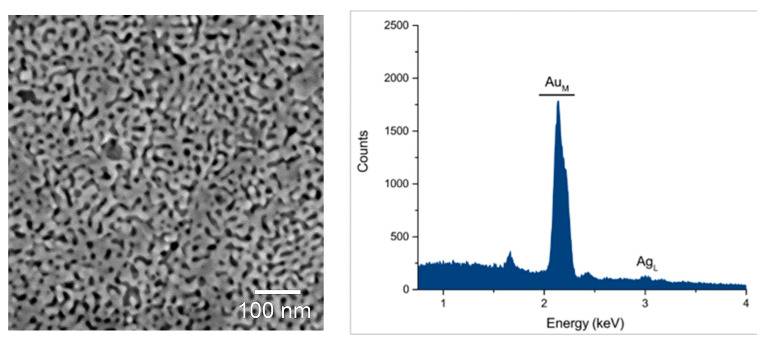
SEM image and EDS spectrum of NP Au after 24 h of dealloying.

The morphology and the composition of the obtained NP Au were investigated by SEM and EDS measurements, respectively. Figure 1 shows the SEM image of the NP Au surface and an EDS spectrum of the examined area. The NP Au highlights a bicontinuous structure of interconnected ligaments and pores with a mean ligament diameter around 15 ± 5 nm and a mean pore diameter around 14 ± 5 nm. Moreover, EDS measurements reveal a residual Ag atomic content around 12%.

**Figure 2:**
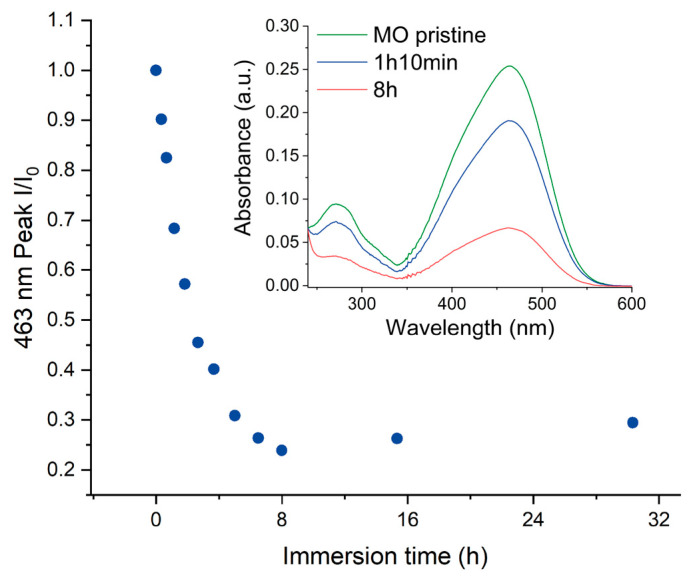
MO discoloration rate. The inset shows the UV-Vis spectrum of MO before NP Au immersion, 70 min and 8 h after the immersion.

In order to investigate the behavior of NP Au with MO, a monolith of this material was immersed into an aqueous solution of the dye with a concentration of 2.0 × 10^−5^ M. The trend over time of this system was monitored by UV-Vis spectroscopy measuring the solution absorption spectrum at different time intervals. Figure 2 illustrates the discoloration rate of the MO solution after the immersion of NP Au, calculated by considering the ratio between the intensity of the MO absorption peak around 463 nm after a certain time of immersion and that of pristine MO solution. The peak intensity decreases with time until 8 h, after which it slightly increases before reaching a constant value around 29% of the initial peak intensity.

The discoloration tests were repeated multiple times, yielding a mean final I/I_0_ ratio of approximately 25% (±5%). The notable standard deviation is likely attributable to the varying surface areas of the NP Au pellets utilized in the tests.

**Figure 3:**
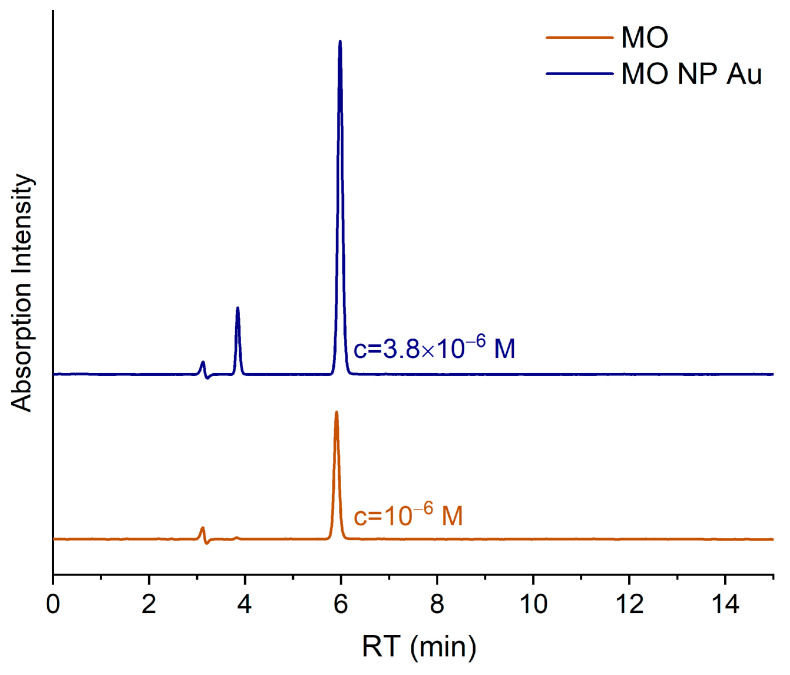
HPLC chromatograms of reference MO solution (1.0 × 10^−6^ M) and of residual solution after NP Au immersion.

HPLC measurements were conducted on the MO solution after 30 h of NP Au pellet immersion (see Figure 3). The chromatogram of a reference solution of pure MO shows a peak at ca. 5.9 min of retention time, whereas that of the residual solution of the above-described discoloration test presents the characteristic peak of MO along with a peak at 3.84 min and a smaller one around 3.1 min.

However, the latter can also be found in the initial MO solution and in MilliQ H_2_O, with comparable intensities. These findings suggest that NP Au can cause MO degradation, as reported by Hakamada et al. [32], found in the initial MO solution and in MilliQ H_2_O, although only partially, in agreement with the UV-Vis data. Moreover, the calculated MO concentration by HPLC is systematically lower than that calculated from UV-Vis measurements. This feature can be ascribed to the superposition of the absorption peak of MO and that of the degradation product (hereinafter referred as DP), which could lead to a concentration overestimation with spectroscopic measurements. Since the peak around 463 nm is characteristic of a N=N bond, it can be supposed that this bond was not broken in the degradation product, in agreement with the results found for the MO photodegradation in the presence of TiO_2_ [44]. Another interesting piece of evidence is that only this peak appears in the HPLC chromatograms of different residual MO solutions, suggesting that the degradation process does not continue after the formation of this compound.

**Figure 4:**
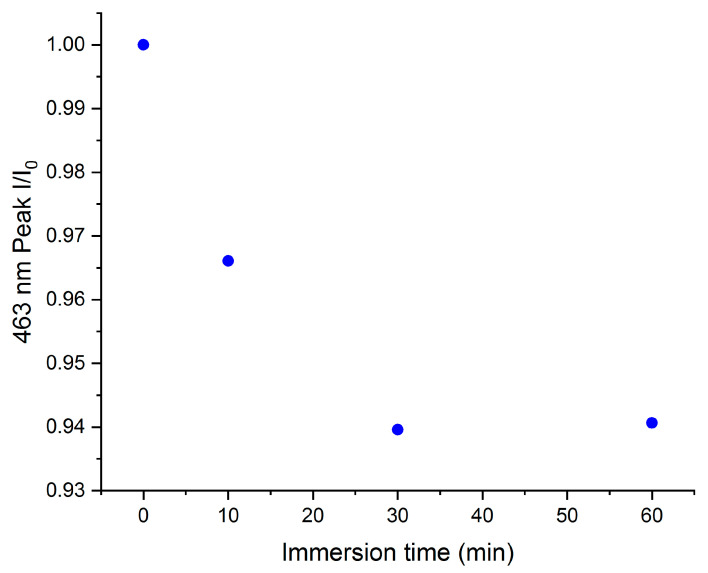
Trend of relative concentration vs. immersion time for NP Au pellet dealloyed for 5 s in order to obtain a thin NP layer.

To evaluate the effect of a reduction in the surface area of gold, an NP Au sample was prepared by treating with HNO_3_ for 5 s only on one face of a AuAg alloy pellet. Since the short time of dealloying induces the formation of only a thin NP layer, a significantly lower surface area can be expected in comparison with the NP Au obtained with 24 h of leaching on both faces. In Figure 4, the discoloration rate when a pellet of NP Au with a thin NP layer was immersed into a solution of MO is reported. It can be observed that the intensity of the peak at 463 nm slightly decreases at the beginning and then reaches a plateau at around 94% of I/I_0_, significantly higher compared to the plateau value measured in the immersion of the pellets porous throughout their entire thickness. This fact points to a dominant non-catalytic process; indeed, in the case of a prevalent catalytic degradation, a slower discoloration rate should be expected (due to a lower active surface area accessible to the reactant molecules), but not such a dramatic change in the final I/I_0_ ratio.

**Figure 5:**
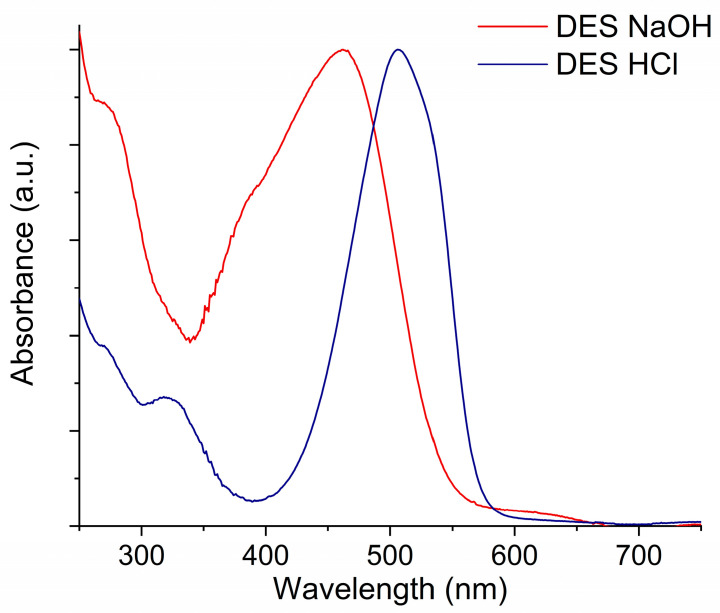
Normalized UV-Vis spectra of solutions desorbed from different NP Au pellets in HCl and NaOH 0.1 M.

Figure S4 shows instead the discoloration rate of the MO solution when an NP Au monolith was repeatedly immersed in fresh solutions at the same concentration. Here, it can be seen that the plateau level (the final I/I_0_ ratio) increases after each immersion, suggesting the establishment of an equilibrium of absorption. Therefore, to have further adsorption, a higher MO equilibrium concentration is needed when more NP Au adsorption sites are occupied. To investigate in depth the nature of this process, the desorption of MO from NP Au pellets was attempted. When the pellet was immersed in water, no observable MO desorption was noticed, while when the pellet was immersed either in NaOH or in HCl, the solution turned colored. The obtained solutions were analyzed by UV-Vis (see Figure 5) and HPLC measurements, which highlight the presence of MO along with other undefined compounds. In the UV-Vis spectra in Figure 5, the characteristic peak of MO is red-shifted when desorbed in HCl and blue-shifted when desorbed in NaOH. This is a typical behavior of MO, by which it is used as a pH indicator; by varying the pH of the solution, the ratio between protonated (red) and deprotonated (yellow) forms of the molecule changes, producing a change in the position and shape of the resulting absorption peak [45]. After the desorption in both solutions, it was observed that the NP Au pellet did not cause MO discoloration anymore, also after having neutralized the surface with the HCl and NaOH for the samples treated with NaOH and HCl, respectively. This finding suggests that an irreversible change occurred to the NP Au surface following its contact with the MO solution.

**Figure 6:**
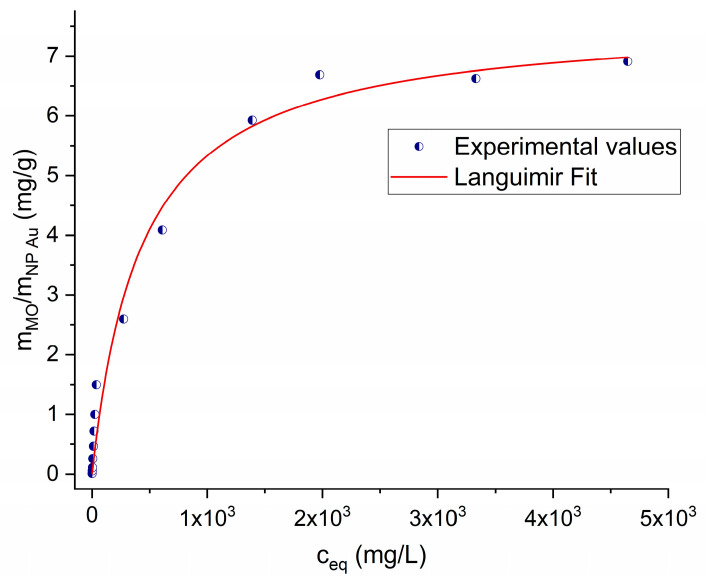
Adsorption isotherm of MO in NP Au pellet.

**Figure 7:**
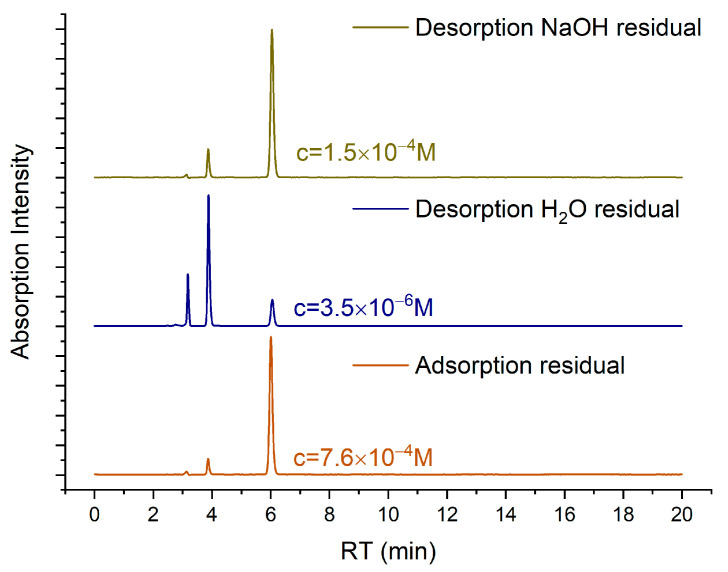
HPLC chromatograms of residual solutions of adsorption and desorption in different concentrations of MO: after adsorption in 10^−3^ M solution (orange), desorption in H_2_O (blue) and desorption in NaOH solution (green).

To clarify the adsorption–desorption mechanism, an adsorption isotherm was constructed by exposing the same NP Au pellet to gradually higher concentrations of the MO solution, from 4 × 10^−6^ M to 1.4 × 10^−2^ M. The adsorption isotherm is shown in Figure 6 and Figure S5 and can be well fitted by the Langmuir adsorption model [46]. The total amount of MO that was lost from the solutions is around 1.6 mg with a pellet of 230 mg. Moreover, after the complete saturation of the sample, desorption was repeatedly induced by exposing the sample to MO solutions with decreasing concentrations and then to pure water. It was estimated that 30% of the MO adsorbed was released. The HPLC measurements shown in Figure 7 clearly indicate that, along with MO, the degradation product found in the residual solutions of the adsorption tests is also present after desorption. In this case, the amount of MO adsorbed on NP Au was so high that it was possible to remove around 50% of the disappeared amount by immersing the pellet multiple times in pure water and then another 5% was extracted during immersions in the NaOH solution. This fact confirms the hypothesis that this compound is also partially adsorbed by NP Au.

**Figure 8:**
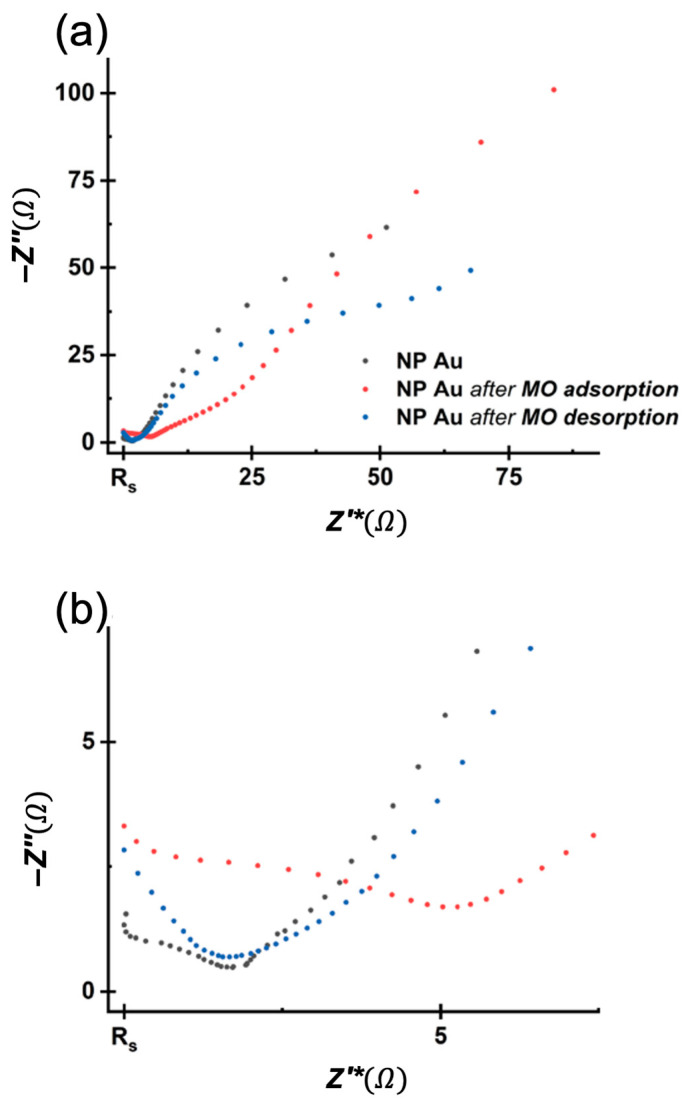
Nyquist plot of NP Au pristine, NP Au after adsorption and NP Au after desorption (**a**) and magnification on the high-frequency region (**b**).

It is still difficult to determine the percentage of MO adsorbed and that of MO degraded because the amount of degradation product seems to be much lower than the amount of MO disappeared and that was not finally desorbed. It is supposed that MO and the degradation product are not completely desorbed from the NP Au surface. With the aim of verifying if the interaction of NP Au with MO causes irreversible changes to the surface and, therefore, whether regeneration of the pellet is possible or not, measurements of electrochemical impedance spectroscopy (EIS, see Scheme S1) and X-ray photoelectron spectroscopy (XPS) were performed. NP Au was analyzed in its original pristine state, after the absorption of the dye, and after its desorption. Figure 8 reports the Nyquist plot of the analyzed samples, each one rescaled for its own solution resistance (Rs) value. As one can see, the electrochemical response varies significantly in all three situations but NP Au after the adsorption differs definitely more than the other two. More specifically, after MO adsorption, the NP Au pellet loses part of its capacitive feature for a more resistive response. Then, after the desorption, the material does not completely regain its original behavior. These observations, although qualitative estimations, can be a hint of non-reversible processes that occur at the surface, such as changes in morphology or some species (MO or some degradation products), which, being hardly desorbed, decrease and modify the active surface of the material.

Some alternatives for the electrochemical characterization of the surface area of NP gold were explored by Rouya et al. [47]. In particular, EIS was used to calculate the double-layer capacitance and, therefore, the estimation of the surface area. Concerning this method, EIS analysis was conducted at the OCP using a 0.1 M solution of HClO_4_, and the dependence of the imaginary part of the impedance on frequency is reported in Figure S6. In a graph of this kind, a log-log slope of –1 in the medium-to-low frequencies region corresponds to typical purely capacitive behavior. Since our cases differ from ideality (with log-log slopes in the range 0.5–0.75), the system should be better described by a constant phase element. Despite the use of this approximation, the slope in the three samples changes in agreement with our previous considerations. Moreover, the slope changes from −0.75 to −0.55 when the surface is modified with the adsorption of MO, whilst it reaches −0.60 after desorption. As already observed, the not complete desorption of the dye and/or the related surface modifications can also be qualitatively highlighted by the partial recovery of the pristine capacitive behavior.

**Figure 9:**
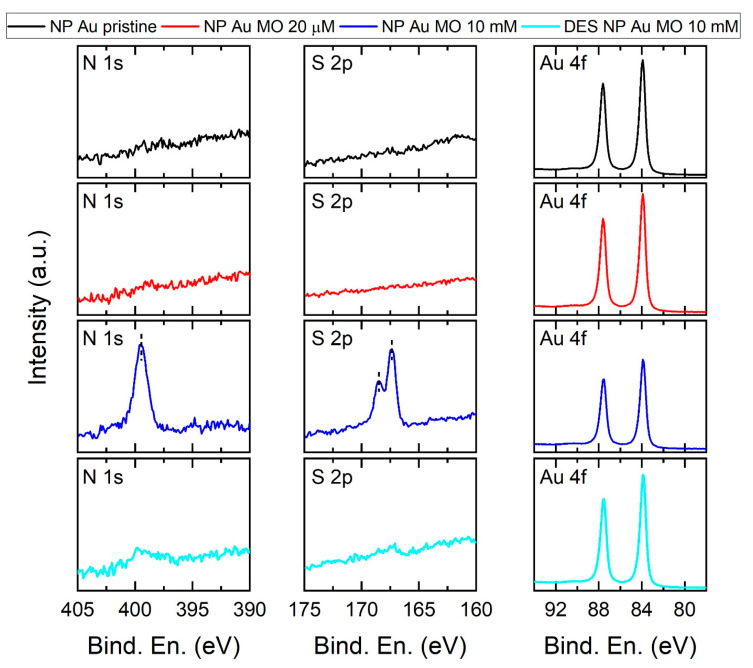
XPS measurements on pristine NP Au (black lines) and three NP Au immersed in MO solutions: 20 µM solution (red lines), 10 mM solution (blue lines) and after complete MO desorption (pale blue lines). The left, middle and right panels report the XPS spectra collected over the energy regions for N 1s, S 2p and Au 4f signals, respectively.

**Figure 10:**
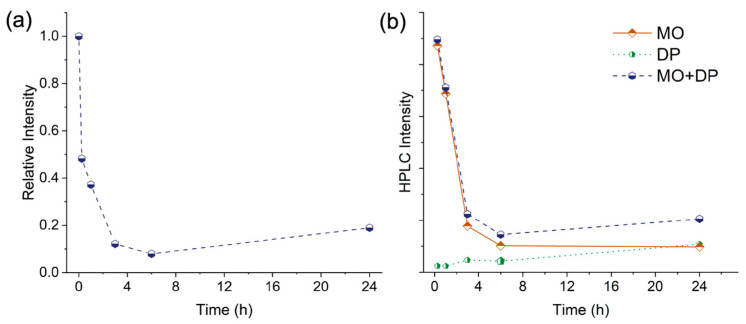
(**a**) UV-Vis relative intensity of absorption peak at 463 nm at different times after NP Au powder immersion; (**b**) HPLC absorption intensities of MO (RT = 6.0 min), DP (RT = 3.9 min) and sum of the two intensities (MO + DP).

XPS measurements were also performed and are reported in Figure 9. A comparison between the pristine sample (in the figure, NP Au pristine) and that obtained after the immersion into the MO solution 2 × 10^−5^ M (NP Au MO 20 mM) does not highlight any important difference. This finding might indicate either not effective adsorption of MO on the Au surface or an extremely low MO content, even lower than the detection limit of this technique. However, the sample NP Au 10 mM (blue line), which was immersed into a more concentrated solution, clearly shows the presence on the Au surface of molecules containing N and S, as indicated by the N 1s peak at (399.5 ± 0.2) eV and by the S 2p_3/2_ and S 2p_1/2_ peaks at (167.3 ± 0.2) eV and (168.5 ± 0.2) eV. The observed XPS signals are consistent with the azo- and sulfonate-moieties of MO, in agreement with literature reports [48]. Moreover, the sample DES NP Au MO 10 mM (pale blue line) obtained by immersion in the same solution, and which underwent desorption until the washing solution was colorless, presents the residual presence of N 1s (at approx. 399.7 eV) and S 2p (at approx. 167.4–168.6 eV) XPS peaks, which can be ascribed to MO and/or its degradation products adsorbed onto the surface of the NP. It is interesting to note that, despite the presence of signals assigned to MO, the position of the peaks corresponding to the Au 4f electrons (namely, the Au 4f_7/2_ peak at 83.9 eV and the Au 4f_5/2_ peak at 87.6 eV) is not affected. The lack of shift of these peaks was put forward by Hakamada et al. as proof of the absence of dye molecules in their NP Au sample after immersion into the MO solution [32]. To further explore the behavior of NP Au, the powder was finally immersed in MO solution, instead of the pellet. In this way, the surface area of NP Au was hugely increased and the percolation time into the pores of the material reduced. As can be seen in Figure 10a, this resulted in a faster discoloration of the MO solution even with a MO/NP Au mass ratio 80-fold higher than that of the test reported in Figure 2. These results show that MO can be nearly completely removed from the solution. However, by prolonging the immersion time, it was observed that the stoppage of the discoloration process was followed by a modest increase in the 463 nm peak intensity. This phenomenon can be clarified by looking at the HPLC measurements in Figure 10b. The MO concentration appears to continuously decrease in the solution while the intensity of the second HPLC peak increases with time. By summing the two peak areas, the same trend was observed through UV-Vis measurements. Therefore, a part of the adsorbed MO is slowly degraded into a second product that is partially desorbed into the solution. This test gives a clear indication of the contribution of adsorption and catalytic phenomena in this system; adsorption is the more relevant and faster phenomenon, which results in the disappearance of 90% of MO in the first 3 h, with a global recollection—i.e., the amount of residual MO after the adsorption and desorption tests—of 87% of the initial MO amount, as can be seen in Table 1. Meanwhile, catalysis plays a secondary role in terms of magnitude and speed, leading to the degradation of less than 37% of MO in 24 h of immersion, an amount that comprises both the degraded and the still adsorbed MO molecules.

## 3. Materials and Methods

### 3.1. Materials

Au powders (99.995%) were purchased from Alfa Aesar, Ag powders (99.9%) from Aldrich, nitric acid (70%) from BDH, and methyl orange powders from Acros Organics. Ultrapure water (18.2 MΩ·cm) was produced with a Milli-Q Millipore (Merck KGaA, Darmstadt, Germany) water purification system.

### 3.2. NP Au Fabrication

The AuAg precursor alloy was prepared by mechanical alloying of Au and Ag powders in 3:7 atomic proportion. A quantity of 2 g of the mixture was placed inside a hardened steel vial with two hardened steel spheres of 8 g each. The powders were milled in a SPEX 8000M (SPEX SamplePrep, LLC, Metuchen, NJ, USA) ball miller for 16 h. NP Au was prepared by cold-pressing the as-prepared AuAg powder into pellets with a diameter of 13 mm and a mass of 350 mg (see Figure S1 in the Supplementary Materials), followed by chemical corrosion in HNO_3_ 70% for 24 h. After the dealloying, the pellets were washed 5 times with MilliQ water and then dried overnight under vacuum (Figure S2).

### 3.3. Immersion Tests in MO Solutions

#### 3.3.1. Pellet Tests

NP Au pellets were immersed in 3.3 mL of a 2 × 10^−5^ M MO solution (0.063 mg MO/g NP Au) in comparable conditions with experiments reported by Hakamada et al. [32]. After immersion, the pellets were washed with ultrapure water, dried, and immersed again in 0.10 M HCl or NaOH solutions for desorption tests. The process was repeated until a negligible amount of MO could be desorbed from the pellets. The experiments were performed both in the dark and under light; however, no differences were observed.

#### 3.3.2. Powder Tests

A quantity of 47 mg of NP Au powder was immersed in 6 mL of 1.20 × 10^−4^ M MO solution (5 mg MO/g NP Au). After immersion, the powder was washed with ultrapure water, dried and immersed again in 0.10 M HCl or NaOH solutions for desorption tests. The process was repeated until a negligible amount of MO could be desorbed from the powder.

### 3.4. UV-Vis Measurements

Electronic absorption spectra were recorded with a UV-Vis spectrophotometer (Agilent Technologies) (Cary Series Spectrophotometer) in a quartz cell of 10 mm path length.

### 3.5. High-Performance Liquid Chromatography

Solutions were analyzed by a 1260 Infinity II (Agilent Technologies, Santa Clara, CA, USA) HPLC system, equipped with a Kinetex (5 µm C18 100 Å, 250 mm × 4.6 mm) column (Phenomenex, Torrance, CA, USA) and a single wavelength UV-Vis absorption detector. Analyses were performed at 40 °C under isocratic conditions, with a mobile phase (flow rate 0.8 mL min^−1^) composed of a mixture (24/76 *v*/*v*) of acetonitrile and a 10 mM ammonium acetate solution. Absorption at 463 nm was recorded.

### 3.6. Scanning Electron Microscopy/Energy Dispersive Spectroscopy

The precursor and dealloyed materials were investigated by scanning electron microscopy (SEM) using a Zeiss (Zeiss, Oberkochen, Germany) Merlin microscope, equipped with a Schottky electron source, operated at an acceleration voltage of 5 kV and at short working distance (<2 mm). Secondary electrons (SE) were collected to provide fine details on the surface morphology and using an in-lens detector. Energy dispersive X-ray spectroscopy (EDS) measurements were carried out in the SEM exploiting an Oxford silicon drift detector (SDD) (Oxford Instruments, Abingdon, UK) with a detection area of 60 mm^2^ and with the microscope working at the same acceleration voltage of 5 kV. Au and Ag standardless quantitative analysis was performed using the Oxford AZTEC 2.1 software.

### 3.7. N_2_ Adsorption–Desorption Isotherms

Textural analysis of an NP Au pellet was carried out with an ASAP 2020 system (Micromeritics, Norcross, GA, USA), by determining the nitrogen adsorption–desorption isotherms at −196 °C (Figure S3). Before analysis, the sample was heated overnight under vacuum up to 200 °C (heating rate, 1 °C/min).

### 3.8. Electrochemical Measurements

The NP Au was characterized by means of electrochemical impedance spectroscopy (EIS) using a 0.1 M HClO_4_ solution in a conventional three-electrode cell in which a saturated calomel electrode (SCE) was the reference and a platinated titanium net was used as the counter electrode. All the EIS measurements were carried out using an AUTOLAB PGSTAT302N (Metrohm, Herisau, Switzerland) potentiostat/galvanostat equipped with the FRA analyzer and controlled with the NOVA software 2.1.6 at the open circuit potential (OCP); the frequency was varied from 63 kHz down to 0.1 Hz with an amplitude of 0.01 Hz.

### 3.9. X-ray Photoelectron Spectroscopy (XPS)

XPS analyses were carried out with a Kratos Axis Ultra^DLD^ (Kratos Analytical, Manchester, UK) spectrometer using a monochromatic Al Kα source operated at 20 mA and 15 kV. Wide-scan analyses were carried out with an analysis area of 300 × 700 µm and a pass energy of 160 eV. High resolution analyses were carried out with the same analysis area and a pass energy of 10 eV over the binding energy regions typical for N 1s, S 2p and Au 4f signals. Spectra were analyzed using CasaXPS software (version 2.3.24).

## 4. Conclusions

In this paper, an investigation was reported on the catalytic activity of NP Au in the degradation reactions of MO, a molecule that belongs to the class of azo dyes. Hakamada et al. reported that NP Au was able to catalyze the complete degradation of MO [32]. Our experiments instead show that the degradation of the dye occurs only partially and that an important part of the MO is adsorbed onto the large surface of the NP material. Indeed, UV-Vis measurements on the solutions obtained from the tests of desorption show that MO, along with the MO degradation product, is released in a large amount by NP Au. Moreover, these findings are also in agreement with the HPLC measurements, which proved that MO with another molecule are present in the same solutions. Furthermore, EIS measurements suggested that the surface of NP Au was modified by the immersion into the MO solution and that, even after a prolonged desorption, it did not recover the pristine conditions. XPS analysis showed the presence of molecules containing N and S atoms on the surface of NP Au both after the immersion and after the desorption procedures, in agreement with the EIS measurements. These results are strengthened by observations made on NP Au powder in MO solution, in which it was possible to distinguish a faster and more important adsorption process—which led to the disappearance of 90% of MO in the first 3 h—from a slower and minor catalytic process, which, together with the irreversible adsorption process on the metal surface, was responsible for less than 40% of the MO disappearance after 24 h of immersion.

## Figures and Tables

**Table 1:** Residual MO fractions in the solution after immersion of NP Au powders for different time intervals and recovered MO fractions of initial MO, recovered both from adsorption and desorption solutions.

Immersion Time (h)	Residual MO (%)	Recovered MO (%)
0.25	48	98
1	37	95
3	10	87
6	6	79
24	5	63

## Data Availability

Data are contained within the article.

## Supplementary Materials

The following supporting information can be downloaded at: https://www.mdpi.com/article/10.3390/molecules29091950/s1, Figure S1: SEM image of the pellet before the chemical corrosion; Figure S2: Schematic representation of the NP fabrication process; Figure S3: N_2_ gas adsorption–desorption isotherm; Figure S4: Relative concentration of MO over time for the same sample repeatedly immersed in fresh MO solution; Figure S5: Comparison between several adsorption models. Scheme S1: Schematic illustration of adsorption and desorption process and related EIS measurements; Figure S6. Log-log dependance of the imaginary part of the impedance vs. frequency and magnitude and fit of the linear part in the medium-to-low frequencies.
