# Supplementary material for: Nanoporous Au Behavior in Methyl Orange Solutions
Source: Molecules. 2024 Apr 24;29(9):1950. doi: 10.3390/molecules29091950 (PMC11085355; doi:10.3390/molecules29091950)
Supplement: Supplementary file 1 [file molecules-29-01950-s001.zip › molecules-2905404-supplementary.pdf]

Supplementary Materials for the Article

Nanoporous Au behavior in methyl orange solutions

Andrea Pinna [1], Giorgio Pia [1,*], Nicola Melis [1], Mirko Prato [2], Maria Giorgia Cutrufello [3,4], Elisa Sogne [5], Andrea Falqui [6] and Luca Pilia [1,*]

[1] Department of Mechanical, Chemical and Materials Engineering, University of Cagliari, Via Marengo 2, 09123 Cagliari, Italy; andrea.pinna94@unica.it (A.P.); nicola.melis87@unica.it (N.M.)
[2] Materials Characterization Facility, Italian Institute of Technology, Via Morego 30, 16163 Genoa, Italy; mirko.prato@iit.it
[3] Department of Chemical and Soil Sciences, University of Cagliari, S.P. Monserrato-Sestu Km 0.700, 09042 Cagliari, Italy; gcutrufe@unica.it
[4] National Interuniversity Consortium of Materials Science and Technology(INSTM), Via Giuseppe Giusti 9, 50121 Firenze, Italy
[5] PoliFAB, Polytechnic of Milan, Via Giuseppe Colombo, 81, 20133 Milan, Italy; elisa.sogne@polimi.it
[6] Department of Physics"Aldo Pontremoli", University of Milan, Via Celoria 16, 20133 Milano, Italy; andrea.falqui@unimi.it
* Correspondence: giorgio.pia@unica.it (G.P.); e-mail: luca.pilia@unica.it (L.P.); Tel.: +39-0706755063 (L.P.)

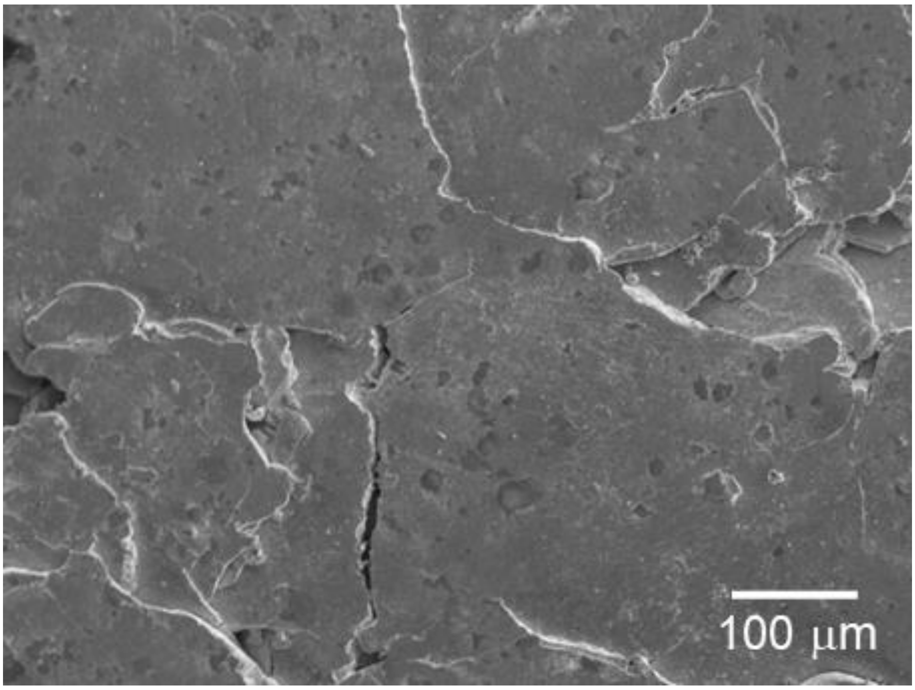

Figure S1. SEM image of the pellet before the chemical corrosion in HNO_3 70% for 24 h.

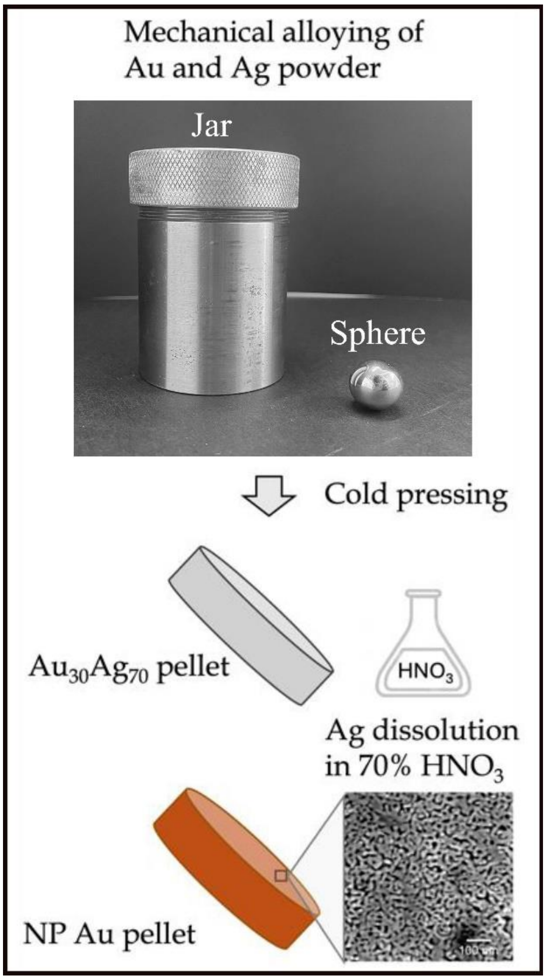

Figure S2. Schematic representation of the nanoporous gold fabrication process.

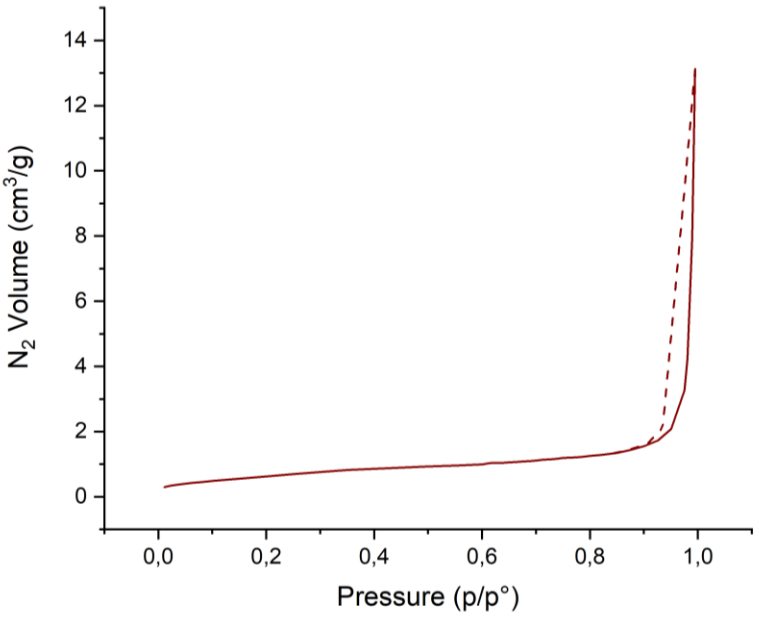

Figure S3. N_2 gas adsorption–desorption isotherm for the nanoporous gold pellet.

Textural analysis of an NP Au pellet was carried out with an ASAP 2020 system (Micromeritics), by determining the nitrogen adsorption–desorption isotherms at −196 °C. Before analysis, the sample was heated overnight under vacuum up to 200 °C (heating rate, 1 °C/min). Surface area was calculated by the Brunauer–Emmett–Teller (BET) equation. The BET analysis based on the measurement reported in Figure S3 gave a value of surface area of roughly 2.5 m^2/g. According to the paper by Wittstock *et al.* [1], in the case of NP Au, this approach could lead to an underestimation of the real surface area of the pristine NP.

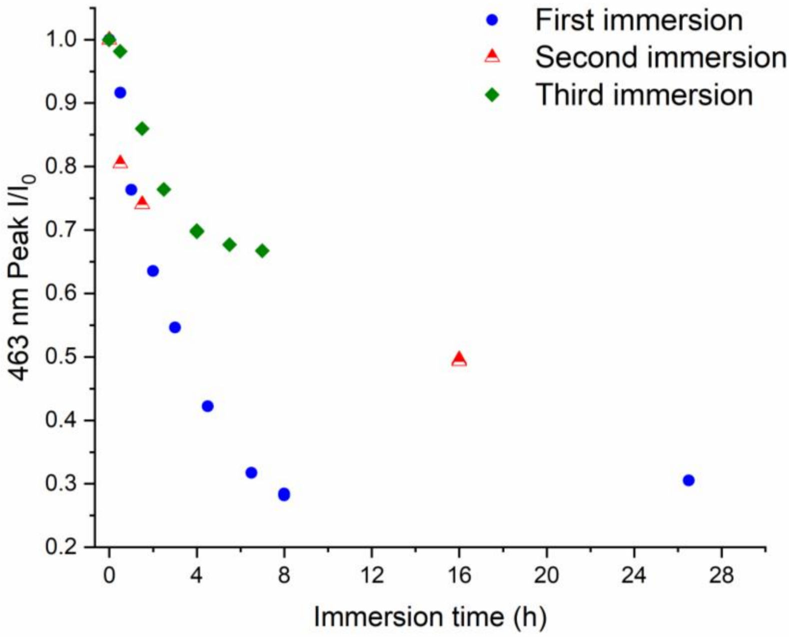

Figure S4. Relative concentration of MO over time for the same sample repeatedly immersed in fresh MO solution.

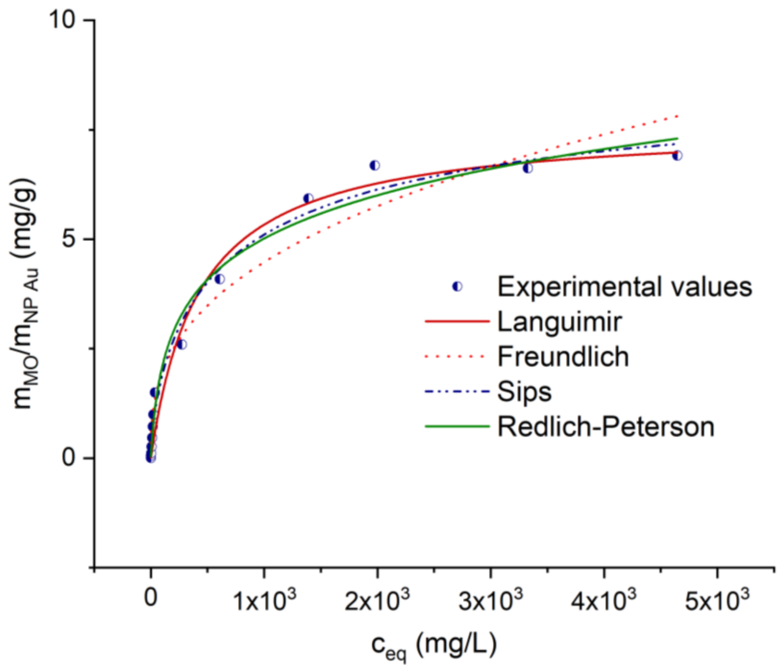

Figure S5. Comparison between several adsorption models. The best fitting is given by the Langmuir one.

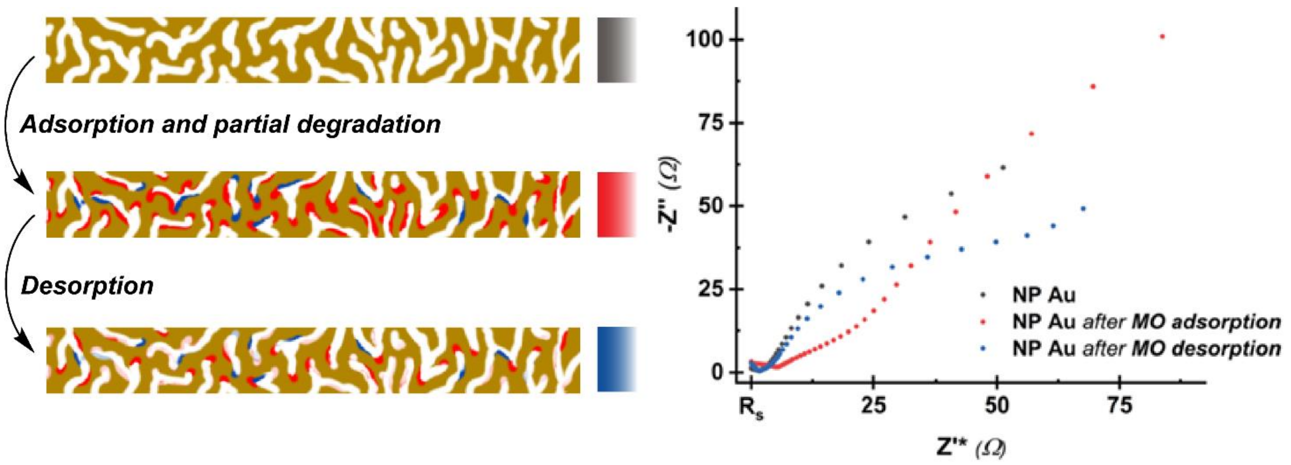

Scheme S1. Schematic illustration of adsorption and desorption process (left), and related Electrochemical Impedance Spectroscopy measurements (right).

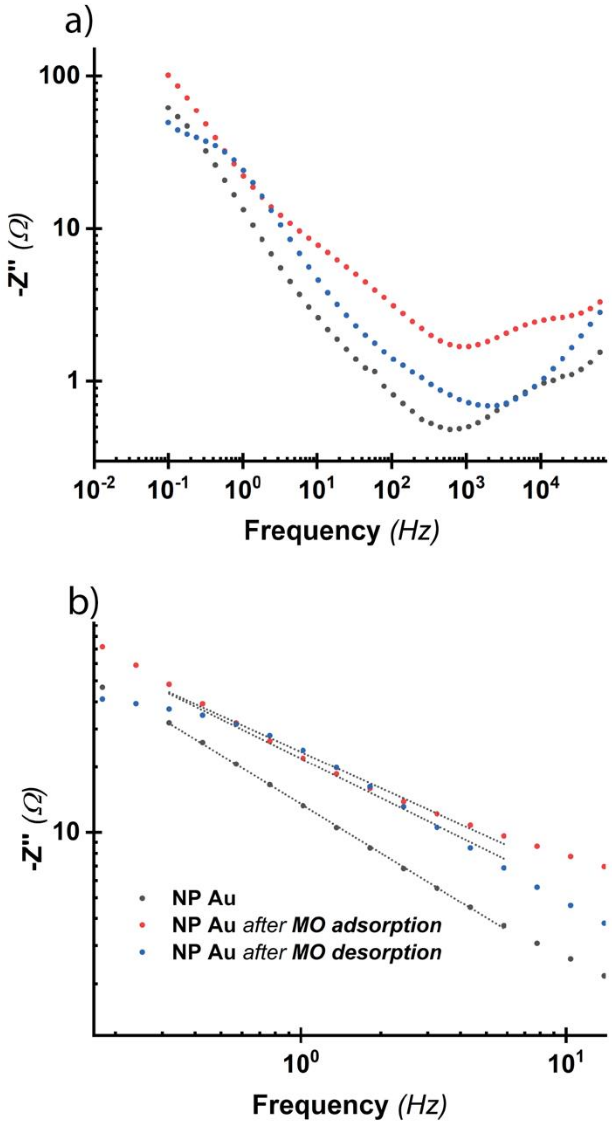

Figure S6. (a) Log-log dependance of the imaginary part of the impedance vs. frequency. (b) Magnitude and fit of the linear part in the medium-to-low frequencies.

Reference

1. Wittstock, G.; Bäumer, M.; Dononelli, W.; Klüner, T.; Lührs, L.; Mahr, C.; Moskaleva, L.V.; Oezaslan, M.; Risse, T.; Rosenauer, A.; et al. Nanoporous Gold: From Structure Evolution to Functional Properties in Catalysis and Electrochemistry. *Chem. Rev.* **2023**, *123*, 6716–6792. https://doi.org/10.1021/acs.chemrev.2c00751.
